# On the Role of Psychological Health and Buoyancy in EFL Teachers' Professional Commitment

**DOI:** 10.3389/fpsyg.2022.897488

**Published:** 2022-06-02

**Authors:** Manman Li

**Affiliations:** School of Foreign Languages, West Anhui University, Lu'an, China

**Keywords:** buoyancy, teachers' professional commitment, psycho-educational environment, EFL teachers, psychological health

## Abstract

Teachers have the main role in cultivating the future members of the community by their effort in the educational space, and indeed, the quality of education would not be accomplished without the ultimate determinations of enthusiastic and committed educators. Henceforth, there is a need for more investigation regarding the conception of teacher commitment and the aspects that affect it. Educator commitment has an important function in influencing educators' presentation; thus, it is necessary to determine the fundamental elements that contribute to educator commitment. Moreover, buoyancy is another fundamental construct in a psycho-educational environment helping people with academic challenges. Psychological health is another term considered as a significant dimension in an academic environment. The present review tries to focus on psychological health and buoyancy that affect educators' job commitment using the theoretical agenda of positive psychology and the sociocognitive theory. Succinctly, this review of the literature has implications for scholars, theorists, and practitioners seeking for inspecting the roles of buoyancy and psychological health on teachers' professional commitment.

## Introduction

Despite educators being mainly employed to teach, they are associated deeply with various assignments aside from in-person instruction, such as curriculum design and content progress, course arrangements, class administration, society relationships, information technology, welfare and security, asset administration, and learners' well-being (Pishghadam et al., 2019). Indeed, the teaching career is frequently characterized as a greatly anxiety-provoking career that can put educators' physical and psychological health at risk (Pepe et al., 2018). Educators encounter the great degrees of psychological exhaustion, mental anguish, and burnout relative to other career categories (Fiorilli et al., 2017; Pishghadam et al., 2019). A bulk of studies in previous years have warned that the psychological stress among educators is on the rise (Moriana and Herruzo, 2004; Fueguel and Montoliu, 2005), which is worsened by high rates of apprehension, hopelessness, and reduced self-confidence that they may also have a negative impact on their work (Matud et al., 2002). The situation is so severe that eight out of 10 educators report that the main disease in the group is psychological issues (Moriana and Herruzo, 2004). Various reasons why educators are exposed to psychological health issues include the large load of work (highly crowded classroom, misconduct, and violence of learners, demands of parents, work assessment, and inspections), inadequate resources (not being recognized for the profession, shortage of materials, incorrect schedule for working, and removal of assistant to help educators), a bad working climate, and tensions between colleagues (Bauer et al., 2006).

However, reliant on the PP progress in education, currently, there has been an increasing scope of the literature on positive emotions in language education (MacIntyre et al., 2016; Wang et al., 2021) and to better clarify educators' mental elements, a great number of experiential investigations have been carried out to examine the connections between several educator-associated factors, such as educator self-effectiveness, exhaustion, emotive intelligence, effectiveness, commitment, and career fulfillment (Skaalvik and Skaalvik, 2007; Viel-Ruma et al., 2010). Instructional commitment is another educator factor that has gained a lot of attention in studies and deals with an educator's amount of mental involvement in the instructional career (Fathi and Savadi Rostami, 2018). When educating teachers, their commitment is defined as their emotional connection to the teaching career, career relations and school, coworkers, learners, and parents (Lee et al., 2011). Furthermore, among all common actions related to work, researchers have the highest concern for commitment. This is because those who work with higher commitment levels are thought to be more likely to achieve institutional and individual-level outcomes, namely, employee turnover, presentation, and willingness to remain or quit the institution (Razak et al., 2010). Educator commitment is a vital phenomenon to comprehend as it is closely related to notions, such as education quality, educator adaptation, educator attendance, educator exhaustion, educator retention, school effectiveness and productivity, school organizational health, learner attitudes, and learning results (Klein et al., 2009). Teachers' commitment is a significant correlate of teachers' performance, and committed teachers have a higher motivation to create positive changes among their learners (Sinclair et al., 2006). Committed teachers not only try hard to foster development among their learners, but also make lots of efforts for their professional development as teachers (Shukla, 2014). As stated by Simpson and Hood (2000), educators' commitment refers to enthusiasm regarding instruction and education, building relationships with learners, exhibiting constructive demeanors toward learners, and being insightful regarding learners' rationales, strong points, demands, and circumstances (Lee et al., 2011).

The requirements of work and non-work functions can cause anxiety; however, the capability of controlling anxiety and poising career and life functions can affect one's demeanor as well as career fulfillment and professional commitment, which mirror workers' commitment to the work (Ballout, 2009). That is, an educator's career commitment has many dimensions and relates to the objects of commitment, namely, the teaching career, school institute, learners, and subject matter (Moses et al., 2017). As it is essential to the expert activities of educators, it has got a great level of consideration in the pedagogical literature, and various predictors of professional commitment are related to educators' well-being and health (Kieschke and Schaarschmidt, 2008).

Indeed, psychological health, considered a concept having many aspects, is understood in terms of the lack of destructive states in addition to the existence of constructive states (Keyes, 2005). Psychological health is theorized as an individual condition that can alleviate the results of disturbing occurrences (surrounding circumstances) for overall health (Veronese and Pepe, 2014). Contrastingly, mental suffering includes common senses of stress, societal flaw (dysphoria), lack of self-esteem, and, more extensively, deconstructive affection (Cocker et al., 2013). Experiencing reality at the beginning of instruction involves a great number of adaptation hardships, an absence of sufficient direction and help mechanisms, and an absence of general fulfillment that results in a decrease in expert commitment (Fernet et al., 2016). The significance of the scenario is better understood, regarding the fact that not any experienced event is shown in the media. Of course, according to the description of WHO (2001) regarding psychological health, educators responsible for elevating youngsters and the youth should know about their abilities, be capable of solving the encountered issues, be productive and effective in their works, and make contributions to the community wherein they live. Thus, establishing settings where psychological health is formed has high importance to raise individuals' psychological health inside the procedure of educator training. As the definition implies, it states effective performance dysfunction and being ill.

Recently, self-determination theory (SDT) has found growing significance and is regularly used in studies regarding positive psychology and welfare (Deci and Ryan, 2000). Based on SDT, meeting an individual's primary psychological wishes will raise their psychological health and reduce depressive symptoms and tension. Therefore, SDT is used in these areas as training in individual growth and health, family, interpersonal relationships, sports, and psychotherapy, and therefore efforts to decide the situations for a healthful individual (Ryan et al., 2008). Consistent with Deci and Ryan (2000), SDT lays the groundwork for comprehending both goal-directed behavior and psychological growth and welfare, and it therefore, explains the reasons and influences of human acts.

Furthermore, detecting the difficulties intrinsic in the ESL/EFL settings, researchers have suggested a novel term for flexibility known as “academic buoyancy.” This alludes to the capability of efficiently dealing with the regular difficulties and hardships of academic life (Comerford et al., 2015). The notion of academic buoyancy has its origins in PP and has a dynamic method to deal with difficulties and hardships in education. As opposed to emphasizing deconstructive aspects and inconveniences, academic buoyancy regards a person's feelings, capabilities, and strong points to elevate his or her health and mental growth (Jahedizadeh et al., 2019). Buoyancy is characterized as a person's self-awareness of their capability of effectively managing the inconveniences of everyday life like difficult assignments (Parker and Martin, 2009). Simply put, it is the ability to withstand and conquer the difficulties, inconveniences, and failures of one's teaching profession (Verrier et al., 2018). Like any other notion, buoyancy is impacted by inner and outer elements. On the one hand, inner elements, such as self-effectiveness, self-esteem, inspiration, and agency, can affect a person's academic buoyancy. On the other hand, outer elements, such as sociocultural settings, academic environments, and shareholders, could also specify a person's academic buoyancy (Comerford et al., 2015).

Grounded on the literature review, in numerous investigations, teachers who were professionally committed seem to be more pleased and fulfilled with their occupations (Basu, 2016; Bashir, 2017). Regarding the significance of commitment to retain educators with high quality, it is important to investigate how and why educators can change their commitment at different phases of their professional experience. To the best of the researcher's knowledge, not enough study has investigated the role of buoyancy and psychological health in the language education domain so far, and their function in augmenting teacher commitment has not been examined. Therefore, it makes an innovative route of inquiry in the arena of EFL teachers' psychology.

## Review of the Literature

### Academic Buoyancy

The definition of buoyancy refers to the ability to adaptively react to the kinds of negligible difficulties commonly encountered during the period (Martin and Marsh, 2009). Academic buoyancy is part of the broaden-and-build theory in PP of positive affections (Collie et al., 2017), which views positive affections as a significant tool for psychological development and enhanced happiness, along with a required goal by itself (Fredrickson, 2001). Based on the broaden-and-build theory, constructive feelings develop an individual's temporary thoughts and actions and increase their long-term personal resources (Fredrickson, 2001). Therefore, the academic buoyancy concept concentrates on people's reactions instead of the existence of everyday difficulties and highlights on proactive instead of reactive approach to these difficulties (Martin and Marsh, 2009). It is assumed to be located on a range and varies based on the personal level and environmental level variables. Building academic buoyancy is tied to several cognate words, namely, flexibility, resistance, and handling all of them have their own precise literal meaning. Thus, a line must be drawn between these terms. Resilience is one of those factors that seem to be on par with buoyancy. However, it varies in reality (Collie et al., 2015). Although the two concepts have the same theoretical underpinnings, resilience has constrained use in academic settings because it fails to address the disadvantages that frequently occur in the academic life of individuals (Phan and Ngu, 2014). These failures and efforts are not always considered difficulties. That is, they are not considered in jeopardy of the growth cycle. Thus, buoyancy is equivalent to daily flexibility (Martin and Marsh, 2009). Moreover, the difference between these two constructs falls into two aspects. The first one asserts that whereas educational resilience pertains to continual under accomplishment, immense sense of tension regardless of continual failure, it pertains to the usual experience of single weak scores or performance, usual levels of anxiety and day by day pressures, and endangering self-confidence due to poor performances. The second one asserts that while resilience applies to the experimental effects (stress and hopelessness), the absence and overall disaffection with school, and complete and objection to educators, academic buoyancy applies to low levels of tension and self-confidence, lower enthusiasm and participation, and managing poor feedback on schoolwork (Martin and Marsh, 2009).

### Psychological Health

One definition provided for psychological health is a state not only featuring the lack of psychological distress signs, but also the existence of further constructive indicators of emotional well-being, also called thriving or flourishing (Su et al., 2014). Such definition is unanimous, though various questions still have not been responded to concerning its basic structure. Studies to date have commonly reinforced the notion that psychological health has many aspects, and the basic aspects can be distinguished according to whether they manifest further global aspects of psychological health or distress (Keyes, 2005; Gonzalez-Roma et al., 2006). There is a significant assumption regarding such models where psychological health and distress are the separate states instead of the final point of a basic range, so that psychological health and distress levels may individually differ from each other within the same individual. As far as we know, this hypothesis was not formally evaluated through methods that adequately separate the alteration attributed to a general psychological well-being concept from the variance recognized to particular aspects of psychological health and distress.

Based on the basic psychological needs (Deci and Ryan, 2000), humans have three intrinsic primary needs: autonomy, competence, and relevance. Such needs are psychological instead of being physiological, i.e., they are emotional and societal, not that of biological needs. It is also essential for survival as they are considered to feed psychological development, integration, and health (Deci and Ryan, 2000). They can be viewed as determining factors of psychological health, as meeting the desires contributes to the effective performance of functions. The necessity for autonomy refers to the independence to define, schedule, and execute individual's manner and lifestyle which is different from independence. A selfhood system linked to an autonomy system and community support is a major factor in determining how to handle stress (Skinner and Edge, 2002). They have a significant role when people have to deal with the problems they face, since each person has his or her own reactions to challenges, difficulties, and events, and these reactions are the result of deliberate actions. People who act autonomously receive the most satisfaction in life and suffer less from emotional issues (Julien et al., 2009). Autonomy refers to the need to be the origin of one's actions and to engage in activities of one's own choice (Ntoumanis et al., 2009).

In addition, competence refers to one's confidence in their aptitude to handle specific conditions (Ryan et al., 2008). People require a large amount of efficiency and buoyancy in doing their tasks, human relations are the origin of happiness and unhappiness, and human happiness and well-being rely on relativeness. A sense of relation affects treating the emotional and communicative difficulties (Deci and Ryan, 2016). Psychological health relies on the satisfaction of three needs (Deci and Ryan, 2000). All the above-mentioned needs help to efficient performance and psychological health, none of which can be denied. In addition, the needs do not contradict each other but complement each other (Sheldon and Bettencourt, 2002). These three primary needs are monotonous global inclinations and are intrinsically available in people and thus do not vary from one value to another (Deci and Ryan, 2000). Satisfaction of the needs that feed the human soul fosters psychological well-being, whereas failing to satisfy them or stopping them, i.e., hunger due to the lack of food, can cause discomfort.

### Teachers' Professional Commitment

Commitment is regarded as a mental connection between a worker and his or her institution, and it alludes to emotive elements, such as attentiveness, beliefs, and the embracement of constructive demeanors, with regard to particular matters (Fernet et al., 2016). Commitment is an element of one's perseverance in higher education, and it is an element of learners' perseverance in higher education (Strauss and Volkwein, 2004). Academic commitment is deemed as a modern area of research inside its large and multi-aspect meaning. Most of the studies in this domain are associated with the communication areas, and structural and academic commitment has only lately been investigated (Viljoen, 2015). The scholastic commitment was at first conceptualized according to the amount of actual endeavor and time spent on the learning and scholastic activities (Viljoen, 2015). However, Human-Vogel contends that the time and endeavor seldom mirror the richness required for the scholastic commitment in the study. He regarded commitment as an aspect of meaning, investment, substitute standard, fulfillment, and degree of commitment (Human-Vogel, 2013). Teaching commitment is critical to mitigating educator turnover, executing curriculum novelties, and introducing discipline changes, keeping program continuity, continued progress, and improving learner growth (Robinson and Edwards, 2012; Han and Wang, 2021). Studies have shown some important variables that affect teaching commitment (e.g., managing class, competence in delivering syllabus, balance in work-life, and problems of organizing class) and emphasizing the necessity of exploring the relationship between various competency domains and teaching commitment (McKim and Velez, 2016).

Teacher commitment is defined by Arjunan and Balamurugan (2013) who stated that it is a zeal for the career of instruction or a particular dimension of instruction. Professional commitment is the intention of being involved with the school and the school society. It is a conviction that educators possess an expert obligation that goes beyond the setting of the class and even the borders of the school. Correspondingly, Obi-Nwosu (2012) declared that educators with great commitment work harder have a stronger attachment to school, and more dedication to teaching aims than those with low commitment. Moreover, organizational commitment is associated with general perception, satisfaction, sense of belonging, recognition of excellence, and interest in a specific organization (Meyer and Allen, 2004). Also, as stated by Wang and Shen (2012), professional commitment is a kind of career commitment that focuses on the significance of work in a person's life. The aspects of this type of commitment include the following: (a) emotional commitment, i.e., the commitment to professional success (career-related emotions), (b) prescriptive commitment, i.e., the commitment that emerges due to strong class standards, and (c) continuous commitment, meaning a commitment to the outcomes of significant loss when someone quits their career.

The meanings of professional commitment vary along a continuum from characterizing commitment based on the manners and the degree to which a person is engaged in particular assignments in their immediate career setting to characterizing commitment empirically and based on the significance of the career in the person's life. The major manners and empirical elements of professional commitment are regarded as focusing on customers, faithfulness, expert independence, and adaptation to expert excellence and morals (Bogler and Somech, 2002). Professional commitment is one of the most significant elements that affect career presentation (Mowday et al., 2013), and this is because the career is an essential component of an employee's life. Professional commitment is characterized as an integral part of the instructing setting as it mirrors the educators' mental and emotive attachment to the career and can portray his or her zeal for academic work (Farkas et al., 2000).

## Pedagogical Implications

This review has some recommendations for EFL educators and teacher instructors in which it cares about educators and specialists in the EFL teaching realm to extend their outlook on the prominence of educator professional commitment and the way to boost it through the significant role of psychological health and buoyancy. The remarkable function of commitment in teachers is undeniable as it is believed that committed educators might possess strong mental connections with their school, learners, or subject matter; furthermore, their behavioral patterns may differ based on the commitments (Cohen, 2000). In addition, committed educators act inside the path of school targets and are keen to spend more effort and time to work to help school development. Likely, after principals mediate, inspire educators to understand their efforts, offer them excellent comments in the course of their job, and attempt to construct proper relationships inside their schools, so they display better dedication in the direction of their career (Nguni et al., 2006). In the same vein, committed educators are optimistic in dealing with difficult instructional circumstances, are more positive about finding answers to educational issues, and have more accountability for their achievements and setbacks. Some past experiential studies have demonstrated that educators' self-effectiveness influences their commitment to instruction. Committed educators can argue that they spend their time on continuing education and do not stop learning new teaching tactics in teaching learners. They are devoted to using any opportunities to challenge themselves and continue learning for their learners' success (Klassen and Chiu, 2010; Canrinus et al., 2012; Wang and Guan, 2020). As a result, policymakers in various learning-related organizations should take into account the role of professional commitment. For instance, the principals of the school as the main decision-making center should encourage educators to attend workshops that guide them to develop their careers through being buoyant. Educators must be strong and academically buoyant and resilient when facing problems, such as cultural inconsistency and language differences, and they should form a linguistic identity while teaching, which in turn leads to caring relationships with students and brings about their commitment and engagement in the class.

As the psychological health of the teacher has a noteworthy function in shaping the educators' commitment, the preparation of helpful settings and situations is believed to be essential in meeting the demands of autonomy, skill, and relevance in teacher training institutes, which will also enhance the psychological health of future educators that accordingly lead to commitment. Autonomous students devote their energy to learning, and when they are skillful, they will strive to overcome learning challenges (Niemiec and Ryan, 2009). When these needs are caught up, the person becomes inactive, reticent, and ill-being which hinders their commitment. Educators begin their education career with an inner commitment to teaching learners or a specific subject, but it typically lasts longer for educators to commit themselves to teaching at a specific organization. Thus, the school organization needs to provide educators with adequate autonomy and support, so that they can feel appreciated and respected (Lee et al., 2011). Moreover, educators can grow a sense of social belonging to a society of educators at a specific organization, which can act as a strong driver to encourage and sustain educator commitment over time.

It is believed that the association between the career commitment of EFL educators and several variables, such as age and sex. helps to gain more knowledge regarding educator's commitment. To this end, additional studies with different study designs may be done to have data related to variables in this study, namely, efficient teaching tactics to offer a comprehensive understanding of the problem. It suggests to use some types of intervention in more studies to clarify the problem in the linguistic context. Future research must be done to carefully scrutinize the interactions between the notions discussed in this article and also demographic factors need to be considered in more studies as they will contribute to expand researchers' knowledge of how gender and teacher experience of educators pertain to the aforementioned constructs.

## Ethics Statement

The studies involving human participants were reviewed and approved by West Anhui University Academic Ethice Committe. The patients/participants provided their written informed consent to participate in this study.

## Author Contributions

The author confirms being the sole contributor of this work and has approved it for publication.

## Funding

This paper was supported by Research on the Key and Difficult Problems in the Construction of First-Class English Majors in Local Applied Undergraduate Universities (Grant No. 2021090105) and Research on the Key and Difficult Problems in the Construction of Firstclass English Majors in Local Application Oriented Undergraduate Colleges Under the Background of New Liberal Arts (Grant No. wxxy2021037).

## Conflict of Interest

The author declares that the research was conducted in the absence of any commercial or financial relationships that could be construed as a potential conflict of interest.

## Publisher's Note

All claims expressed in this article are solely those of the authors and do not necessarily represent those of their affiliated organizations, or those of the publisher, the editors and the reviewers. Any product that may be evaluated in this article, or claim that may be made by its manufacturer, is not guaranteed or endorsed by the publisher.
